# Gout Augments the Risk of Cardiovascular Disease in Patients With Psoriasis: A Population-Based Cohort Study

**DOI:** 10.3389/fimmu.2021.703119

**Published:** 2021-07-15

**Authors:** Zhiyong Chen, Yiwen Xu, Miao Chen, Ran Cui, Yu-Hsun Wang, Sheng-Ming Dai, James Cheng-Chung Wei

**Affiliations:** ^1^ Department of Rheumatology and Immunology, Shanghai Jiao Tong University Affiliated Sixth People’s Hospital, Shanghai, China; ^2^ Department of Medical Research, Chung Shan Medical University Hospital, Taichung, Taiwan; ^3^ Institute of Medicine, College of Medicine, Chung Shan Medical University, Taichung, Taiwan; ^4^ Department of Allergy, Immunology & Rheumatology, Chung Shan Medical University Hospital, Taichung, Taiwan; ^5^ Graduate Institute of Integrated Medicine, China Medical University, Taichung, Taiwan

**Keywords:** gout, psoriasis, cardiovascular disease, epidemiology, population based cohort study

## Abstract

**Objective:**

Patients with psoriasis (PsO) have a high frequency of concomitant gout and increased risk of cardiovascular diseases (CVD). We aimed to estimate the synergistic impact of gout on the risk of CVD in patients with PsO.

**Methods:**

A population-based cohort of patients registered in the National Health Insurance Research Database of Taiwan between 2000 and 2013 was stratified according to the presence of PsO and gout. Propensity score analysis was used to match age and gender at a ratio of 1:4. Cox proportional hazard models and subgroup analyses were used to estimate the hazard ratios (HRs) for CVD adjusted for traditional risk factors. The Kaplan–Meier method was used to plot the cumulative incidence curves.

**Results:**

Patients with combined PsO and gout (n = 97), PsO alone (n = 388), gout alone (matched, n = 388) and matched controls (n = 388) were identified. Compared with the patients with PsO alone, the patients with combined PsO and gout had a significantly higher risk of CVD (relative risk 2.39, 95% CI 1.56 to 3.65). After adjustment for traditional risk factors, the risk of CVD was higher in patients with gout alone (HR 2.16, 95% CI 1.54 to 3.04) and in patients with combined PsO and gout (HR 2.72, 95% CI 1.73 to 4.28).

**Conclusions:**

Gout augments the risk of CVD independently of traditional risk factors in patients with PsO.

## Introduction

Psoriasis (PsO) is a common systemic chronic inflammatory disease. It is estimated that approximately 125 million people worldwide have PsO (1). In addition to cutaneous involvement, patients with PsO have an increased prevalence of concomitant psoriatic arthritis (PsA), hyperuricemia, dyslipidemia, hypertension, diabetes, stroke, coronary heart disease, acute myocardial infarction, obesity and depression (1). A recent study found that the standardized mortality rate of PsO is significantly elevated when compared with the general population, and that circulatory disease is one of the leading causes of death in patients with PsO (2). Gout is one of the most prevalent forms of inflammatory arthritis worldwide. It is generally accepted that gout is caused by hyperuricemia and the deposition of monosodium urate crystals in and around joints (3). Similar to PsO, patients with gout also have high frequencies of hypertension, type 2 diabetes, dyslipidemia, cardiac diseases (including coronary heart disease, heart failure and atrial fibrillation), stroke, chronic kidney disease and obesity (3).

The high prevalence of hyperuricemia and gout in patients with PsO has been documented in several observational studies (4–6) and has been confirmed recently by a large prospective cohort study from the USA, in which Merola et al. found that PsO is associated with an increased risk of subsequent gout, with a multivariate hazard ratio (HR) of 1.71. The risk of gout was substantially increased among those with PsO and concomitant PsA, with an HR of 4.95 when compared with participants without PsO (7). Thus, it is rational to speculate that gout or hyperuricemia may augment the risk of developing cardiovascular diseases (CVD) in patients with PsO. However, to the best of our knowledge, the impact of gout on the incidence of CVD in patients with PsO remains unknown. In particular, it has not been investigated in a large prospective cohort study.

To address this issue, we investigated the association between gout and the incidence of CVD in patients with PsO in a population-based, matched cohort study using the National Health Insurance Research Database (NHIRD) with a population of 1 million and 14 years of follow-up.

## Patient and Methods

### Data Sources

This is a population-based prospective cohort study. Data were obtained from NHIRD. The NHIRD enrolls approximately 99% of the 23 million beneficiaries in Taiwan and contains data including diagnoses, drug prescriptions, inpatient care, outpatient visits, emergency hospitalization and diagnoses. The study was approved by the Institutional Review Board of Chung Shan Medical University Hospital.

### Study Participants and Matching

**Figure 1:**
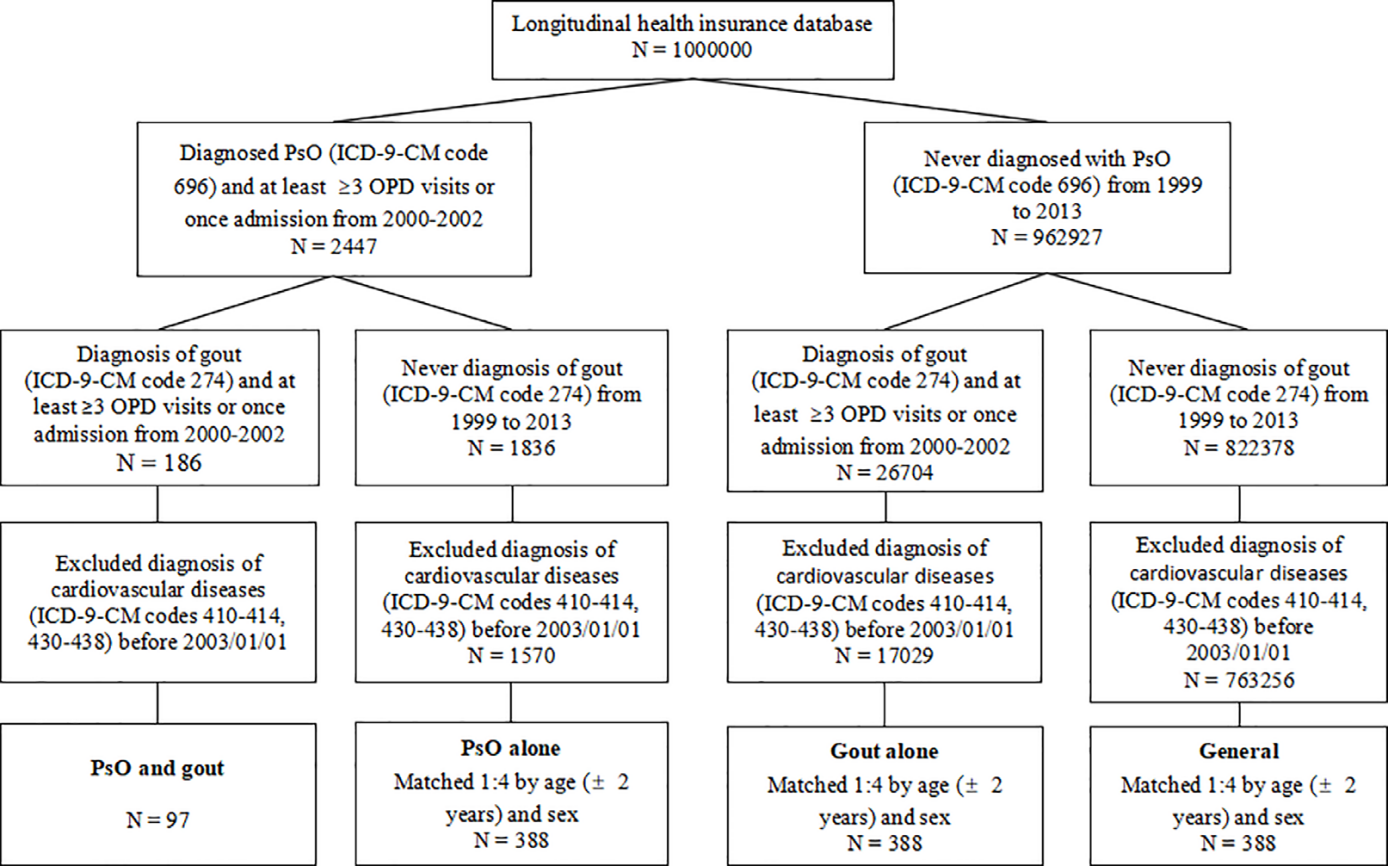
Flow chart of the study design. PsO, psoriasis; OPD, outpatient department; ICD-9-CM, International Classification of Diseases, Ninth Revision, Clinical Modification.

As shown in **Figure 1**, the longitudinal health insurance database (LHID) consisting of data from 1 million individuals was compiled from 2000 to 2013. Diagnoses were identified according to the International Classification of Diseases (ICD), Ninth Revision, Clinical Modification (ICD-9-CM) codes. Between January 1, 2000 and December 31, 2002, 2447 patients were diagnosed as having PsO (ICD-9-CM code 696) in the LHID. This code has also been used in previous population-based epidemiologic study of PsO (8). In the general clinical practice in Taiwan, the diagnoses of PsO, gout and CVD were made at least once by the specialist. Among these patients, 186 were diagnosed as having gout (ICD-9-CM code 274). Patients receiving at least three outpatient visits or one hospitalization were considered as having PsO and gout. The index date was set as January 1, 2003. Patients diagnosed as having CVD (ICD-9-CM code 410 to 414 for ischemic heart diseases; 430 to 438 for cerebrovascular diseases) before the index date were excluded. Finally, 97 patients were included in the group of patients with combined PsO and gout. Follow-up started on the index date and ended at CVD occurrence, the date of withdrawal from the national insurance system or the end of the study (December 31, 2013). New cases of CVD were identified from the database using the records of ICD-9-CM codes mentioned above, with at least three outpatient visits or one hospitalization.

In each group of patients with PsO alone, patients with gout alone, and the general population (persons without PsO or gout), 388 individuals were matched for sex and age (± 2 years) at the index date to minimize potential confounding effects.

### Covariates

The following comorbidities and medications were included in the multivariate analysis to identify the independent risk factors for the incidence of CVD: hypertension (ICD-9-CM codes 401 to 405); hyperlipidemia (ICD-9-CM codes 272.0 to 272.4); chronic liver diseases (ICD-9-CM code 571); chronic kidney diseases (ICD-9-CM code 585); diabetes (ICD-9-CM code 250); chronic obstructive pulmonary diseases (COPD; including ICD9-CM code 491 for chronic bronchitis, ICD-9-CM code 492 for emphysema, ICD-9-CM code 496 for chronic airway obstruction, not elsewhere classified); autoimmune diseases (including ICD-9-CM code 710 for diffuse diseases of connective tissue, ICD-9-CM code 714 for rheumatoid arthritis and other inflammatory polyarthropathies, ICD-9-CM code 720 for ankylosing spondylitis and other inflammatory spondylopathies); arrhythmia (ICD-9-CM codes 426 to 427); and the use of aspirin (defined as at least 30 days of use during the study period). The comorbidities were defined as those receiving a diagnosis within three years before the index date and associated with at least three outpatient visits or one hospitalization.

### Statistical Analysis

The chi-square or Fisher exact test for categorical variables and Student’s *t-*test for continuous variables were used to compare the demographic data between groups. A Kaplan–Meier analysis was performed to assess the cumulative incidence of CVD and a log-rank test was used to test the significance. A Cox proportional hazard model was used to estimate the HR for CVD between groups. A *p*-value < 0.05 was considered statistically significant. Analyses were performed using SPSS software (version 18.0; SPSS Inc., Chicago, IL, USA).

## Results

Compared with general population, participants with PsO alone, with gout alone, and with combined PsO and gout had higher prevalence of hypertension, hyperlipidemia, chronic liver diseases, chronic kidney diseases, diabetes, COPD, autoimmune diseases, arrhythmia and aspirin use (**Table 1**).

**Table 1:** Baseline characteristics of the study population according to the history of psoriasis and gout.

	General (n = 388)	Gout alone (n = 388)	PsO alone (n = 388)	PsO and gout (n = 97)
Age				
<40 years, n (%)	116 (29.9)	91 (23.5)	96 (24.7)	25 (25.8)
40-64 years, n (%)	200 (51.5)	222 (57.2)	216 (55.7)	54 (55.7)
≥65 years, n (%)	72 (18.6)	75 (19.3)	76 (19.6)	18 (18.6)
Mean ± SD	49.2 ± 15.5	50.6 ± 14.9	50.1 ± 15.5	50.3 ± 15.5
Sex				
Female, n (%)	32 (8.2)	32 (8.2)	32 (8.2)	8 (8.2)
Male, n (%)	356 (91.8)	356 (91.8)	356 (91.8)	89 (91.8)
Hypertension, n (%)	28 (7.2)	110 (28.4)	54 (13.9)	31 (32.0)
Hyperlipidemia, n (%)	7 (1.8)	70 (18.0)	19 (4.9)	20 (20.6)
Chronic liver diseases, n (%)	19 (4.9)	69 (17.8)	44 (11.3)	22 (22.7)
Chronic kidney diseases, n (%)	0 (0.0)	11 (2.8)	1 (0.3)	3 (3.1)
Diabetes, n (%)	15 (3.9)	53 (13.7)	29 (7.5)	12 (12.4)
COPD, n (%)	10 (2.6)	27 (7.0)	24 (6.2)	7 (7.2)
Autoimmune diseases, n (%)	1 (0.3)	17 (4.4)	11 (2.8)	11 (11.3)
Arrhythmia, n (%)	2 (0.5)	14 (3.6)	5 (1.3)	0 (0.0)
Aspirin use, n (%)	21 (5.4)	51 (13.1)	25 (6.4)	15 (15.5)

COPD, Chronic obstructive pulmonary diseases; PsO, psoriasis.

The number of patients who developed CVD during the follow-up period is shown in **Table 2**. The incidence density (ID) was 14.83 (95% confidence interval (CI) 11.41 to 19.27) per 1,000 person–years (PY) in the general population, and the patients with gout alone had a 2-fold increased ID compared with patients with PsO alone (36.3/1,000 PY and 18/1,000 PY, respectively). The patients with combined PsO and gout had the highest ID (42.99/1,000 PY; 95% CI 30.40 to 60.80). Using the general population as a reference, the relative risk (RR) of the development of CVD was 2.44 (95% CI 1.78 to 3.36) in patients with gout alone and 2.90 (95% CI 1.88 to 4.48) in patients with combined PsO and gout. In comparison with patients with PsO alone, the combination of PsO and gout was associated with a 239% increase in the relative risk of CVD.

**Table 2:** Incidence density of cardiovascular diseases among different groups.

	Patients	Person-years	Patients with cardiovascular diseases	ID (95% CI)	Relative risk (95% CI)	
General	388	3775	56	14.83 (11.41 to 19.27)	Reference	
Gout alone	388	3254	118	36.26 (30.28 to 43.43)	2.44 (1.78 to 3.36)	
Pso	485	4296	96	22.34 (18.29 to 27.29)	1.51 (1.08 to 2.09)	
PsO alone	388	3552	64	18.02 (14.10 to 23.02)	1.21 (0.85 to 1.74)	Reference
PsO and gout	97	744	32	42.99 (30.40 to 60.80)	2.90 (1.88 to 4.48)	2.39 (1.56 to 3.65)

ID, incidence density (per 1,000 person–years); CI, confidence interval; PsO, psoriasis.

**Figure 2:**
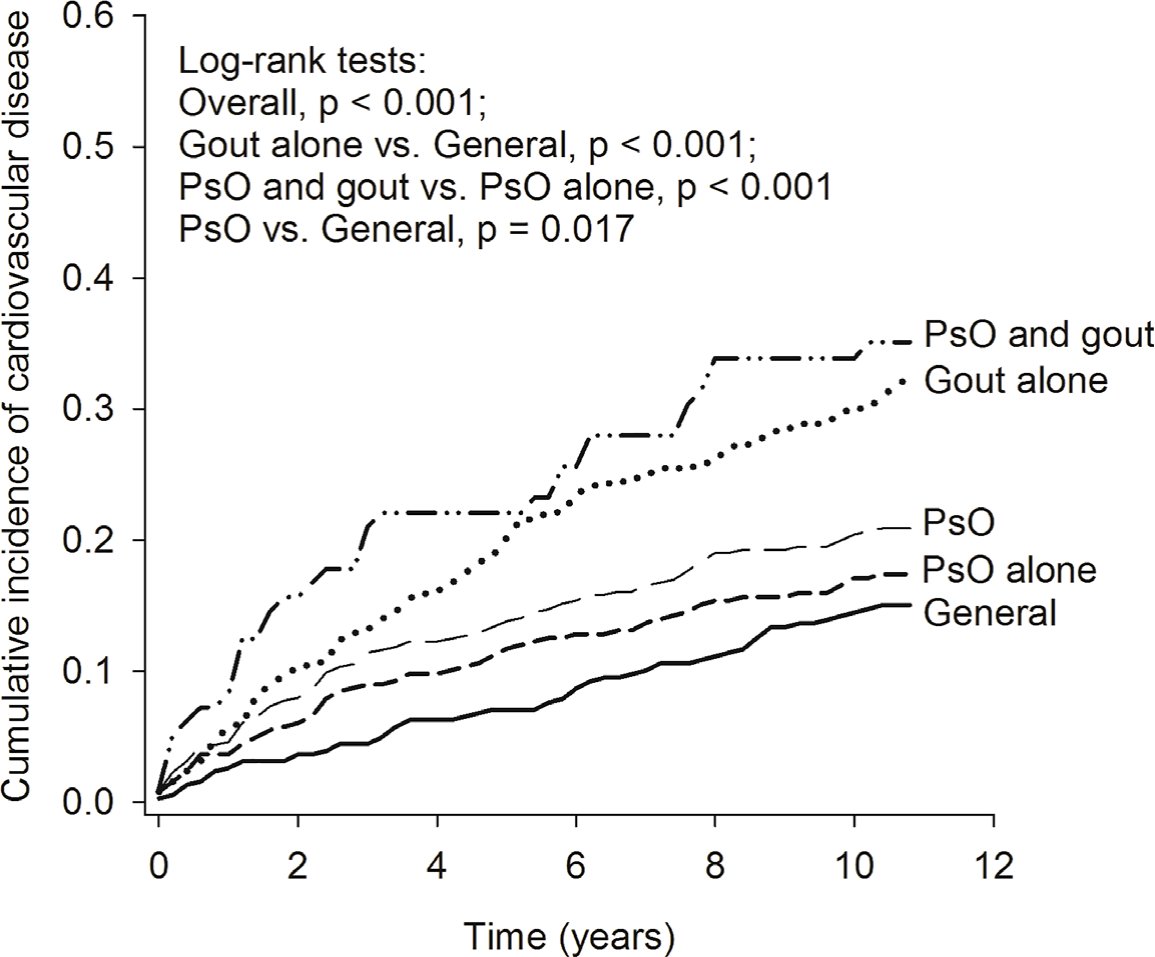
Kaplan–Meier curve of cumulative incidence of cardiovascular diseases in the study groups. PsO, psoriasis.

The Kaplan–Meier curves (**Figure 2**) revealed that the cumulative incidence of CVD increased gradually in the general population, in patients with PsO alone, in patients with PsO (with or without gout), in patients with gout alone, and in patients with combined PsO and gout (log-rank test, overall *p* < 0.001). The risk of CVD in patients with PsO alone tended to be increased but there was no significant difference compared with the general population. The cumulative incidence of CVD was significantly higher in patients with PsO (with or without gout) and in patients with gout alone than in the general population (*p* = 0.017 and *p* < 0.001, respectively). Moreover, the cumulative incidence of CVD in patients with combined PsO and gout was greatly increased when compared with patients with PsO alone (*p* < 0.001).

In the Cox proportional hazard model, the multivariate-adjusted HRs for CVD were increased, ranging from 1.15 (PsO alone) to 2.72 (PsO and gout). The multivariate adjusted HR appeared to be higher among patients aged 40–64 years (HR = 2.80; 95% CI 1.82 to 4.30) and among patients aged ≥ 65 years (HR = 5.68; 95% CI 3.55 to 9.07) than among patients aged 39 years or younger. The presence of hypertension (HR = 1.93; 95% CI 1.45 to 2.58) and COPD (HR = 1.61; 95% CI 1.06 to 2.45) were also associated with increased risk of CVD (**Table 3**).

**Table 3:** Cox proportional hazard model analysis for risk of cardiovascular diseases.

	Univariate		Multivariate^†^	
	HR (95% CI)	*p* value	HR (95% CI)	*p* value
Group				
General	Reference		Reference	
Gout alone	2.40 (1.74 to 3.29)	<0.001	2.16 (1.54 to 3.04)	<0.001
PsO alone	1.21 (0.84 to 1.73)	0.299	1.15 (0.80 to 1.65)	0.462
PsO and gout	2.81 (1.82 to 4.34)	<0.001	2.72 (1.73 to 4.28)	<0.001
Age				
<40	Reference		Reference	
40 to 64	3.17 (2.08 to 4.84)	<0.001	2.80 (1.82 to 4.30)	<0.001
≥65	7.43 (4.77 to 11.56)	<0.001	5.68 (3.55 to 9.07)	<0.001
Sex				
Female	Reference		Reference	
Male	0.89 (0.59 to 1.35)	0.588	0.79 (0.52 to 1.21)	0.280
Hypertension	3.25 (2.52 to 4.18)	<0.001	1.93 (1.45 to 2.58)	<0.001
Hyperlipidemia	1.37 (0.95 to 1.98)	0.095	0.70 (0.47 to 1.04)	0.074
Chronic liver diseases	1.03 (0.72 to -1.48)	0.874	0.88 (0.60 to 1.28)	0.495
Chronic kidney diseases	3.01 (1.34 to 6.76)	0.008	1.31 (0.57 to 3.04)	0.523
Diabetes	2.29 (1.65 to 3.19)	<0.001	1.34 (0.95 to 1.90)	0.096
COPD	2.97 (2.00 to 4.39)	<0.001	1.61 (1.06 to 2.45)	0.026
Autoimmune diseases	1.16 (0.60 to 2.26)	0.659	0.77 (0.39 to 1.51)	0.445
Arrhythmia	2.95 (1.52 to 5.74)	0.001	1.22 (0.60 to 2.48)	0.587
Aspirin use	1.60 (1.13 to 2.28)	0.008	0.78 (0.53 to 1.14)	0.204

HR, hazard ratio; COPD, Chronic obstructive pulmonary diseases; PsO, psoriasis.

^†^Adjusted for age, sex, hypertension, hyperlipidemia, chronic liver diseases, chronic kidney diseases, diabetes, COPD, autoimmune diseases, arrhythmia and aspirin use.

We further performed a subgroup analysis stratified by age and sex to investigate the impact of the presence of concomitant gout on the incidence of CVD in patients with PsO (**Table 4**). The HRs were adjusted for age, gender, hypertension, hyperlipidemia, chronic liver diseases, chronic kidney diseases, diabetes, COPD, autoimmune diseases, arrhythmia, and aspirin use. In the subgroup of age < 65 years, the patients with gout alone (aHR = 1.99; 95% CI 1.31 to 3.01) and patients with combined PsO and gout (aHR = 2.85; 95% CI 1.66 to 4.88) had a significantly higher risk of CVD than the general population. In the subgroup of age ≥ 65 years, patients with gout alone (aHR = 2.73; 95% CI 1.45 to 5.13) and patients with PsO alone (aHR = 2.00; 95% CI 1.10 to 3.64) had a higher risk of CVD than the general population. Among women, a higher risk of CVD was observed in patients with gout alone (aHR = 6.16; 95% CI 1.56 to 24.39) and in patients with PsO alone (aHR = 3.99; 95% CI 1.16 to 13.69). Among men, we also observed a significantly higher risk of CVD in patients with gout alone (aHR = 2.12; 95% CI 1.49 to 3.03) and in patients with combined PsO and gout (aHR = 2.96; 95% CI 1.86 to 4.72) than in the general population. However, the risk of CVD in male patients with PsO alone was comparable with that in the general population.

**Table 4:** Subgroup analysis of risk for cardiovascular diseases.

	Patients	Patients with cardiovascular diseases	aHR^†^ (95% CI)	*p* value
**Age < 65**				
Group				
General	316	38	Reference	
Gout alone	313	82	1.99 (1.31 to 3.01)	0.001
PsO alone	312	32	0.82 (0.51 to 1.31)	0.400
PsO and gout	79	25	2.85 (1.66 to 4.88)	<0.001
**Age ≥ 65**				
Group				
General	72	18	Reference	
Gout alone	75	36	2.73 (1.45 to 5.13)	0.002
PsO alone	76	32	2.00 (1.10 to 3.64)	0.023
PsO and gout	18	7	2.23 (0.91 to 5.49)	0.081
**Female**				
Group				
General	32	4	Reference	
Gout alone	32	11	6.16 (1.56 to 24.39)	0.010
PsO alone	32	10	3.99 (1.16 to 13.69)	0.028
PsO and gout	8	0	NA	NA
**Male**				
Group				
General	356	52	Reference	
Gout alone	356	107	2.12 (1.49 to 3.03)	<0.001
PsO alone	356	54	1.03 (0.70 to 1.51)	0.894
PsO and gout	89	32	2.96 (1.86 to 4.72)	<0.001

aHR, adjusted hazard ratio; PsO, psoriasis; NA, not applicable.

^†^Adjusted for age, gender, hypertension, hyperlipidemia, chronic liver diseases, chronic kidney diseases, diabetes, chronic obstructive pulmonary diseases, autoimmune diseases, arrhythmia and aspirin use.

## Discussion

In this prospective, population-based, matched cohort study, we confirmed that the presence of PsO (with or without gout) and the known risk factors such as age, hypertension and COPD were associated with an increased risk of CVD. The presence of PsO alone increased the risk of CVD in females and individuals aged ≥ 65 years, but not in males or individuals aged < 65. Importantly, we found that the ID for CVD was highest in patients with combined PsO and gout (42.99/1,000 PY). In comparison with patients with PsO alone, the combined presence of PsO and gout was associated with a 239% increase in the relative risk of CVD.

It is well known that PsO is associated with an increased risk of major adverse cardiovascular outcomes such as myocardial infarction (9–12) and stroke (13, 14), and is associated with an increased cardiovascular mortality (15). PsO is also an independent risk factor for coronary artery calcification (16). A high prevalence of metabolic syndrome (MetS) components such as diabetes, hypertension, hyperlipidemia and obesity has been observed in patients with PsO in a previous cross-sectional study (17). These traditional risk factors are generally considered to be the causes of the increased incidence of CVD in patients with PsO. However, the risk of CVD for patients with PsO has not been fully investigated in any large prospective cohort study and has not been analyzed in detail by adjusting for the traditional risk factors in East Asians. In our study, the presence of gout alone was associated with the risk of CVD in all subgroups and in multivariate analyses after adjustment for traditional risk factors. However, the association between the presence of PsO alone and CVD was not statistically significant in multivariate analyses after adjusting for traditional risk factors, and was seen only in females and in individuals aged ≥ 65 years. The discrepancy between the present study and previous studies (9–14) might be explained by the differences in ethnicity, outcomes and confounders included in the analysis. To date, gout or hyperuricemia has not been included in the definition of MetS (18). Our results indicate that there are at least two independent categories of risk factor for CVD in patients with PsO: gout and the components of MetS.

Hyperuricemia is the critical mechanism of the pathogenesis of gout. Various factors can influence serum uric acid (UA) levels, and the serum UA level itself is closely related to conditions such as dyslipidemia, obesity, hypertension, and impaired glucose metabolism, which may contribute to CVD pathophysiology (19). The mechanisms by which hyperuricemia is involved in the pathogenesis of CVD may include but are not limited to the following findings. First, hyperuricemia may induce endothelial dysfunction, which is a key pathophysiological trigger in atherosclerosis. Endothelial cells secrete a number of vasodilators such as nitric oxide, prostaglandin I-2, and endothelium-derived hyperpolarizing factor. It has been found that hyperuricemia can induce human umbilical vein endothelial cell apoptosis and endothelial dysfunction through endothelial nitric oxide synthase (eNOS) phosphorylation and endoplasmic reticulum stress, reducing eNOS activity and nitric oxide production, activating NF-κB, and increasing the levels of inflammatory cytokines (20, 21). It has been suggested that the beneficial effect of allopurinol on CVD may rely more on its ability to reduce oxidative stress than on its impact on urate levels (22). Second, hyperuricemia can increase reactive oxygen species production and inhibit insulin-induced glucose uptake by increasing the phosphorylation of insulin receptor substrate 1 and inhibiting the phosphorylation of Akt. As a result, hyperuricemia can inhibit insulin signaling, induce insulin resistance and accelerate the process of atherosclerosis (23, 24). Third, hyperuricemia may participate in the pathogenesis of CVD by activating the inflammasome pathway. Several studies have shown that UA activates the nod-like receptor protein 3 inflammasome and induces interleukin-1β release in monocytes, macrophages, vascular smooth muscle cells, and endothelial cells (25–28). Other pathogenic roles of UA in CVD may include induction of an imbalance in macrophage M1/M2 polarization (29), HDL dysfunction (30) and increasing the risk of developing high LDL cholesterol and hypertriglyceridemia (31). Several studies have revealed a significant correlation between serum UA levels and increased carotid intima–medial thickness among patients with PsA (32–34).

In the subgroup analyses, we found that the presence of gout alone was associated with increased risk of CVD in all subgroups, with the highest HR (6.16) in females. Although the statistical power may have been insufficient owing to the relatively small number of females, our results suggest a stronger impact of gout/hyperuricemia on the risk of CVD in females than in males. Although the mechanism underlying the impact of gender on the association between UA and CVD remains elusive, our findings are consistent with the previous studies. Fang et al. found that the association between serum uric acid levels and cardiovascular mortality was more robust in women than in men and persisted regardless of traditional cardiovascular risks. They also found that there was no association of serum UA with cardiovascular mortality among men at high traditional cardiovascular risk (35). Hoieggen et al. found also that the association between serum UA and cardiovascular events was stronger in women than in men, with or without adjustment of Framingham risk score (36). An earlier study revealed that an increased serum UA level was an independent predictor of all-cause and heart disease mortality for woman only. Women with a serum UA level of ≥ 416 μmol/L had an almost 5-fold higher risk of ischemic heart disease mortality than those with a level < 238 μmol/L (37).

We found that the presence of COPD was associated with an increased risk of CVD both in univariate analysis (HR = 2.97) and in multivariate analysis (HR = 1.61), which is in line with previous reports. It has been reported that patients with COPD have a 2–3-fold increased risk of CVD compared with age-matched controls after adjustment for tobacco smoking (38). Patients with COPD often have hypoxia during exercise, high resting heart rates, impaired vasodilatory capacity and peripheral, cardiac and neurohumoral sympathetic stress, which may cause CVD. Mechanistically, cytokines involved in the pathogenesis of COPD, such as interferon-γ, interleukin (IL)-1, tumor necrosis factor-α, and IL-6, may contribute to the development of CVD (39). As a well-known risk factor for COPD, smoking is also prevalent in patients with PsO. Our results suggest that the management of COPD and recommendation of smoking cessation may be important for CVD prevention in patients with PsO.

Several guidelines and recommendations for the management of common comorbidities of PsO have been developed (40). However, most of them focus on comorbidities such as MetS, inflammatory bowel disease, psychologic dysfunction and cancer. The management of gout or hyperuricemia is not included in the latest guideline for the care of comorbidities of patients with PsO (41). In addition, a low purine diet was not included in recent dietary recommendations for adults with PsO or PsA (42). A pharmaco-epidemiological study revealed that allopurinol decreased the risk of myocardial infarction by 20% (43). The significant impact of gout on the risk of developing CVD found in the present study strongly suggests that targeted education and therapeutic intervention of gout/hyperuricemia may help to reduce the risk of CVD in patients with PsO, and should be included in the guidelines or recommendations for PsO in the future. Cardiovascular risk is consistently increased in multiple types of arthritis including rheumatoid arthritis, gout, PsA and osteoarthritis (12). Cardiovascular risk algorithms developed for the general population are not accurate in these patients (44, 45). Our study suggests that a PsO specific cardiovascular risk score is needed, in which the presence of gout/hyperuricemia should be included.

The advantages of the present study include that the study involved a population-based prospective design. The large number of participants with a relatively long follow-up period allowed us to perform matching and subgroup analysis to adjust for several possible confounders.

The limitations should also be addressed. First, despite that in the general clinical practice in Taiwan, the diagnoses of PsO, gout and CVD were made at least once by the specialist in LHID, we cannot rule out completely the over- or under-diagnoses of these diseases. Second, owing to the relatively small size of patients with combined PsO and gout, several potential confounding factors such as the use of medications (e.g. allopurinol, statins, anticoagulants and other cardiovascular medications) were not adjusted. Third, laboratory data such as the values of erythrocyte sedimentation rate, C-reactive protein and serum UA were unavailable in the NHIRD. Therefore, the associations between the levels of UA and disease activity of PsO and the risk of CVD could not be investigated in the present study, although it is reported that the association between PsO and metabolic syndrome increases with increasing disease severity of PsO (46). The CVD related life style factors such as smoking, diet preference and body mass index were also not analyzed because of the lack of relevant data.

In summary, our prospective, population-based matched cohort study revealed that concomitant gout is an independent risk factor for CVD in patients with PsO. In addition to control of the traditional CVD risk factors, physicians should consider relevant education and management of gout/hyperuricemia in the care of patients with PsO.

## Data Availability Statement

The raw data supporting the conclusions of this article will be made available by the authors, without undue reservation.

## Ethics Statement

The studies involving human participants were reviewed and approved by Chung Shan Medical University Hospital (IRB, CS15134). Written informed consent for participation was not required for this study in accordance with the national legislation and the institutional requirements.

## Author Contributions

S-MD and JC-CW conceptualized the research. ZC, YX, MC, RC and JC-CW interpreted the data and drafted the manuscript. Y-HW performed data analysis and graph generation and critically revised the manuscript. All authors contributed to the article and approved the submitted version.

## Conflict of Interest

The authors declare that the research was conducted in the absence of any commercial or financial relationships that could be construed as a potential conflict of interest.
